# Estimating Bus Cross-Sectional Flow Based on Machine Learning Algorithm Combined with Wi-Fi Probe Technology

**DOI:** 10.3390/s21030844

**Published:** 2021-01-27

**Authors:** Ting-Zhao Chen, Yan-Yan Chen, Jian-Hui Lai

**Affiliations:** Department of Beijing Key Laboratory of Traffic Engineering, Beijing University of Technology, Beijing 100124, China; ctzlzhao@163.com (T.-Z.C.); cdyan@bjut.edu.cn (Y.-Y.C.)

**Keywords:** Wi-Fi probe, bus passenger flow estimation, feature extraction, machine learning, strain function

## Abstract

With expansion of city scale, the issue of public transport systems will become prominent. For single-swipe buses, the traditional method of obtaining section passenger flow is to rely on surveillance video identification or manual investigation. This paper adopts a new method: collecting wireless signals from mobile terminals inside and outside the bus by installing six Wi-Fi probes in the bus, and use machine learning algorithms to estimate passenger flow of the bus. Five features of signals were selected, and then the three machine learning algorithms of Random Forest, K-Nearest Neighbor, and Support Vector Machines were used to learn the data laws of signal features. Because the signal strength was affected by the complexity of the environment, a strain function was proposed, which varied with the degree of congestion in the bus. Finally, the error between the average of estimation result and the manual survey was 0.1338. Therefore, the method proposed is suitable for the passenger flow identification of single-swiping buses in small and medium-sized cities, which improves the operational efficiency of buses and reduces the waiting pressure of passengers during the morning and evening rush hours in the future.

## 1. Introduction

As a city develops, the increase in the number of cars in use can cause serious traffic congestion in the commercial and core areas. The conflict between the demand for private transportation and the capacity provided by the road network is increasing in many cities. It is therefore important to solve traffic congestion issues by vigorously developing and optimizing a public transportation system. The optimal scheduling of a traditional bus service relies on personal experience, origin–destination (OD) estimation, passenger flow forecasting, and other related factors. There is usually a lack of real-time data for passengers inside and outside the bus, which results in an unbalanced vehicle transportation capacity and reduces the effectiveness of the public transportation system. The advent of smartphones and popularity of Wi-Fi have enabled data to be collected using information technology in the study of traffic behavior. 

The traditional methods of collecting passenger flow information include video detection, manual statistics, and integrated circuit (IC) card [1,2]. The video detection and manual statistics are laborious and expensive because they need real-time updates, and most ticketing systems on buses record when passengers get on a bus, but do not record when they get off the bus. Wi-Fi probe technology collects the media access control (MAC) address and signal strength information of mobile terminal equipment used to analyze the characteristics of passenger flows over a small range and has the advantage of real-time data transmission. Most researchers used this technique to achieve static positioning. For instance, Salamah et al. [3] used a principal component analysis (PCA) to improve the performance and reduce the computational cost of a Wi-Fi indoor positioning system, which was based on machine learning. Some researchers have applied this technique to public transportation research. For instance, Zhang [4,5] proposed the Wilocator method of bus location and predicted bus arrival times. There are many authors who have explored traffic problems by capturing Bluetooth signals, such as Reiff [6], who placed a Bluetooth sensor on a section of road to track the travel path of a vehicle and obtained the OD distribution of the road network at a low cost. Campana [7] applied Bluetooth to indoor navigation. Abedi [8,9] reported that a Wi-Fi probe could detect a larger volume of sample data than Bluetooth and proposed that the Wi-Fi probe data be used to measure the travel time and motion characteristics of pedestrians and bicycles. The data were found to be accurate over a small range.

Currently, bus route passenger flow information is mainly based on video recognition and ticketing data. Video recognition is prone to large errors when determining passenger flows in crowded areas. Fleuret [10] and Takala [11] devised a method to collect changes in passenger flows in a subway station. Fleuret [10] proposed a method for tracking passenger paths in complex environments with POM global optimization based on video capture technology for accurate positional evaluation. Takala [11] used color, texture, and motion information on a surveillance video to track the motion trajectory of an object under different conditions. Hsu et al. [12] used deep learning to identify bus passenger flow based on surveillance video. In addition, researchers in this field have also used many algorithms to analyze surveillance images. Zhu et al. [13] estimated passenger flow based on convolutional neural networks in public transportation systems. Sindagi [14] summarized recent advances in CNN-based single image crowd counting and density estimation. However, it is difficult to obtain the number of passengers in the bus with a single swipe ticket function. Yuwei Li [15] divided the bus stations into two categories according to the nature of land use. Through sampling survey, the probability of bus passengers getting off at each type of station was calculated. By fusing AFC and AVL data of The Hague, the Netherlands, Menno Yap [16] directly determined the exact route and vehicle each passenger used and deduced the stop-to-stop vehicle occupancy for each individual vehicle trip. Therefore, video recognition and ticketing data have limitations in analyzing the flow of bus passengers.

In terms of passenger flow forecast, Myrvoll et al. [17] connected the Wi-Fi probe to the computer for investigation and used one Wi-Fi probe for the sniffing device. The position of the Wi-Fi probe easily leads to the signal strength of the user outside the bus being stronger than that inside the bus and mistakenly identifies users outside the bus as users inside the bus. It is effective to avoid this problem by installing multiple Wi-Fi probes and extracting signal features. Tang et al. [18] proposed method performs best only when the target station has origin-destination smart card transaction records. There are a lot of studies on short-time OD prediction, which is greatly affected by time and space and individual selection behavior; especially, the increase in traffic assignment work due to transfer stations. Therefore, it is necessary for the bus of single-swipe card to obtain the passengers directly.

The detection used in this paper is based on the detection of Wi-Fi or Bluetooth information transmitted by mobile devices. The detector typically detects a large number of unique (MAC) address. Based on data characteristics, classifier classifies the mobile device as being on the bus or outside the bus. Algorithms to identify passengers based on mobile device detection must consider several challenges. One is that the number of mobile devices that can be detected does not correspond to the number of passengers on the bus. Any one passenger may carry several devices, or none at all. It depends on country, age and career. Another challenge is that the signal received outside the bus is stronger than inside, due to factors including the placement of detection device and the number of passengers on the bus. A third challenge is that the wireless signal varies depending on the state of the device (e.g., if it is in use or in sleep mode), on the type of device (e.g., tablet or smartphone), and on the vendor. 

There is also a problem of privacy theft, since the detection device can get the MAC address of the phone. Today, people are sensitive about outside ads being pushed to their phones and they are wary of revealing their personal information. Therefore, our detection device only intercepts the time MAC address and signal strength when acquiring data in the background. To protect privacy, some mobile devices generate a fake MAC address for the phone. Early test results show the randomly generated MAC address of a phone in a new hexadecimal order. This paper did not track the user’s trajectory, but only determined whether the user was in the bus during the fine-grained period. Therefore, the fake MAC address did not affect our subsequent analysis. Many researchers have attempted to use data mining technology to obtain the section passenger flow characteristics of bus lines, which would support line operation and scheduling, fully realize the potential of traditional public transportation networks, improve the service quality of public transportation, and increase the use of public transportation. Therefore, it is of great practical significance to identify passengers inside and outside of bus based on Wi-Fi probe technology.

In the current study, six probes were arranged for the No. 52 bus in Beijing, China, to collect data during the evening rush hour in order to extract the appropriate wireless signal features of users. This paper explored three supervised algorithms of machine learning to learn the rule of label data. The differences between the verify accuracy of various algorithms were compared. In addition, because the data collected by Wi-Fi probe is not a full sample, a correction function to correct the recognition results of the machine learning algorithm were put forward and evaluated.

## 2. Techniques and Methods

### 2.1. Equipment

Wi-Fi probe technology refers to the detection technology based on Wi-Fi or Bluetooth to identify the mobile terminal devices (such as mobile phones, laptops, tablets, etc.) near the wireless access point that have Wi-Fi or Bluetooth enabled. The probe is able to identify the user’s information without the user having access to Wi-Fi. When a mobile terminal device enters the probe signal coverage area and Wi-Fi or Bluetooth function is turned on, it can be detected by the probe. Both iPhone operating systems and Android systems can easily detect and retrieve the device’s MAC address. The collected information includes time, MAC address, type, and signal strength of the access device.

**Figure 1:**
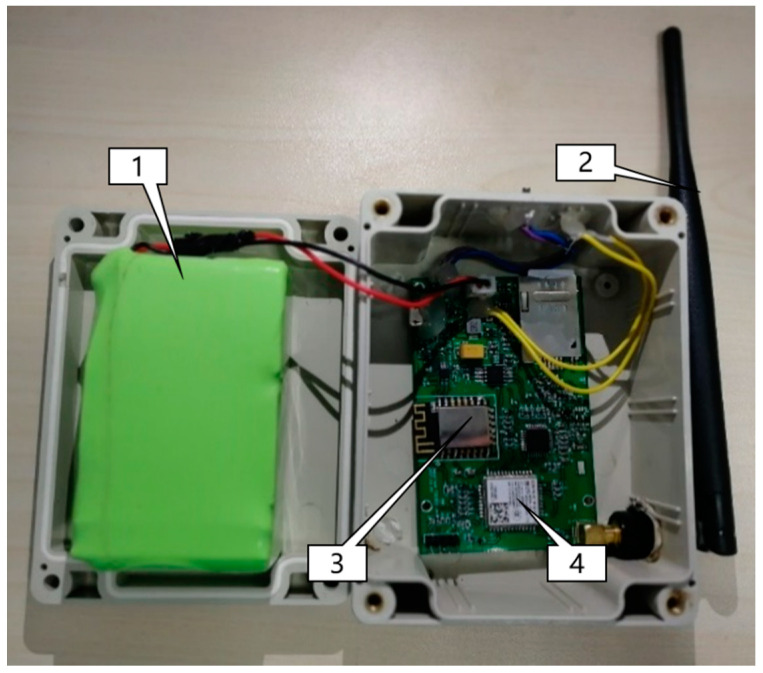
The Wi-Fi media access control (MAC) address scanning hardware used for data collection: battery (**1**), antenna (**2**), Wi-Fi surface scanning chip (**3**), Bluetooth surface scanning chip (**4**).

The size of the probe assembled box is 5 × 7 × 4 cm (Figure 1), and its important components include Bluetooth surface scanning chip, Wi-Fi surface scanning chip, battery and antenna. The device installs a communication card to keep it running and transmits data information to the background through listening software. The scanning accuracy of the device is 1~3 m. When the Wi-Fi button of the terminal device is open, signal information from the Wi-Fi terminal device is obtained between 0~100 m. The scanning frequency of Bluetooth is 300 ms, and that of Wi-Fi is 250 ms. 

**Figure 2:**
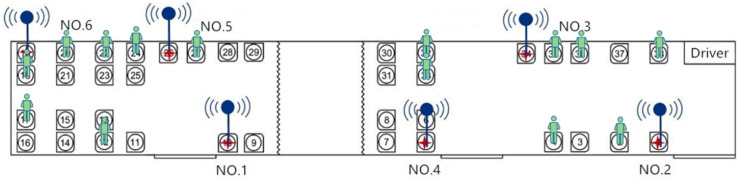
The location of the Wi-Fi probes and volunteers in the bus.

In order to collect the Wi-Fi probe data of passengers during bus travel, our experiment was carried out on Bus 52 in Beijing, China. The purpose of the experiment was to find out the characteristic rules of signal strength inside and outside the bus, and to provide the basis for the later algorithm training and learning. At the same time, a manual survey was used to get the real number of passengers in the bus, so as to prepare for the later check. The whole experiment is divided into two parts:

In the first part, the survey was conducted on 18 January 2019. We recruited 20 volunteers to carry mobile phones and act as passengers in the bus, and collected their data to describe the signal characteristics of passengers in the bus. A total of six volunteers with Wi-Fi probes were seated in bus seats numbered 1, 5, 10, 19, 26, 34 (red four-pointed star in Figure 2), while the remaining volunteers were seated in seats numbered 2, 4, 12, 17, 18, 20, 22, 24, 27, 32, 33, 35, 36, and 38 (green person in Figure 2). The location of Wi-Fi probes and volunteers were evenly distributed, which is to make the collected label data more comprehensively represent the passenger data in the bus. This survey began at the start of 52 bus lines, which allowed volunteers to sit in designated seats.

**Figure 3:**
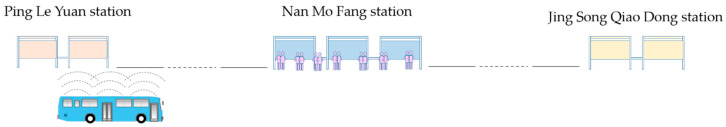
The location volunteers out of the bus.

In the second part, we also recruited twenty volunteers, among whom fourteen volunteers collected their signal data as people waiting at the bus station. In this part of the survey, the six volunteers with Wi-Fi probes who seated at the red four-pointed star (such as Figure 2) were required to take buses to cycle between the three bus stops (such as Figure 3), and the other volunteers were scattered at the second bus station. One of the volunteers on the bus recorded the time they took the bus to leave from the first bus station and to arrive at the third. The three bus stations are Ping Le Yuan station, Nan Mo Fang station, Jing Song Qiao Dong station, respectively. In this part of the survey, it is worth mentioning that volunteers could take any bus that could pass through the three bus stations. When the bus that with Wi-Fi probes passes the Nan Mo Fang station, it collects the signals of the volunteers waiting on this bus station (Figure 3). Then, after the bus had pulled out 100 m, the volunteers walked to the other Nan Mo Fang station across the road and waited for a bus with Wi-Fi probes. According to this model, we collected 5 laps of data.

### 2.2. Dataset

**Figure 4:**
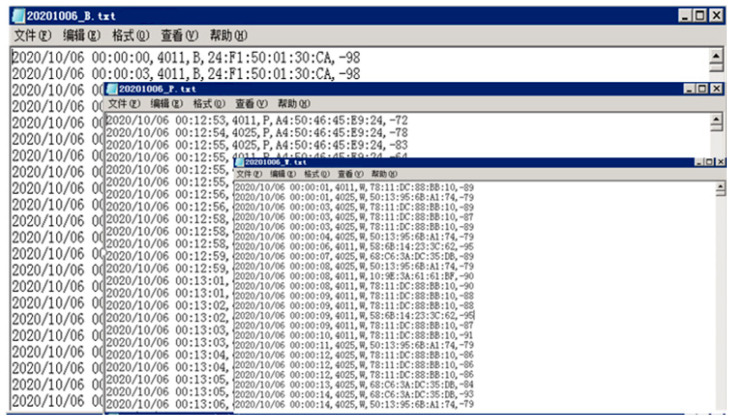
The text format of B, P and W for storing data.

Based on the data MAC type, we use the Java language to divide the data into three types: B, P, and W. Where B is the Bluetooth data type of the Shared bicycle, P is the Bluetooth data type of the mobile phone, and W is the Wi-Fi data type of the mobile phone. Such as Figure 4, the first column is the time the packet was uploaded, which is consistent with the network time. The second column is the number of Wi-Fi probes. In Figure 2, we have simplified the probe numbering, which does not affect the subsequent analysis. The third column is the type of signal: Wi-Fi signal of telephone, Bluetooth signal of telephone, and Bluetooth signal of shared bikes. The fourth column is MACID of users, and the last column is their signal strength.

In order to ensure the accuracy of passenger flow count, the most important part is to filter noise data. A common basis for filtering is the presence of fake MAC. In the process of passenger flow acquisition, our data is obtained by detecting frames. However, in order to protect people’s privacy, some cell phone manufacturers will use random MAC addresses during the scanned stage. Our methodology does not depend on identifying a specific user, but rather a device over some short time-span. This paper is to identify user inside and outside the bus, even if the user uses a fake MAC address, which does not affect our results to a certain extent. For incomplete data (such as the data bar that is missing or added fields due to background data transfer, and those with garbled characters and missing partial contents of the field), they were filtered out. The total data collected during the two parts of the experiment were distributed as shown in Table 1. It can be seen that the data collection by probe No. 4 was limited in the in-bus test, but the remaining probes collected more than 20,000 data points. More than 20,000 data points were collected by each probe in the out-of-bus test and, therefore, the stability of the equipment was considered acceptable.

### 2.3. Feature Selection 

The data characteristics of different time periods were analyzed to identify passengers inside and outside the bus. The original data were divided into periods of 3 min, 2 min, 1 min, 30 s, and 20 s, and the number of labels were 117, 160, 235, 375, and 434, respectively. Due to the number of labels in the 3 min, 2 min, 1 min, and 30 s periods being smaller, the 20 s period was used in the subsequent analysis. 

#### 2.3.1. The Average Signal Strength Detected during the Fine-Grained Time Period (RSSI_AVG)

**Figure 5:**
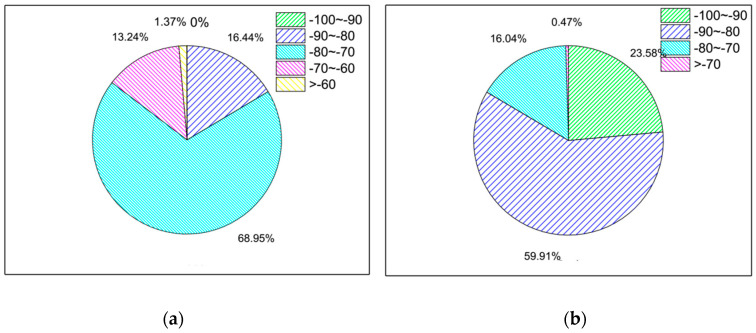
The pie distribution diagram of the average signal strength of the passengers during the fine grain time. (**a**) In the bus. (**b**) Out of bus.

Based on labeled data, the original data information is transformed into features by numerical methods for the model to learn. In this system, as all we know is that the Wi-Fi probe picks up the wireless signals sent by the mobile device over a period of time. Because wireless signal transmission environment is very complex, it is very difficult to directly observe the signal law, especially in the case of bus movement, and the road environment is relatively complex. The signal strength is stronger when it receives the device of the passenger in the bus, but weaker when it receives the mobile device outside the bus. Therefore, it is first considered to construct the Wi-Fi probe to receive the average signal strength of the mobile device during the fine grain time period. The results are shown in Figure 5.

The RSSI_AVG of the in-bus data was mainly distributed in the −80~−70 category, while the RSSI_AVG of the out-bus data was mainly distributed in the −90~−80 category. The proportion of in-bus data was larger than that of out-bus data in the −80~−70 and >−70 categories, while the proportion of out-bus data was larger than that of in-bus data in the −100~−90 and −90~−80 categories. Overall, the signal strength in the out-bus environment was smaller than that in the in-bus environment.

#### 2.3.2. The Standard Deviation of the Signal Strength Detected during the Fine-Grained Time Period (RSSI_ST)

**Figure 6:**
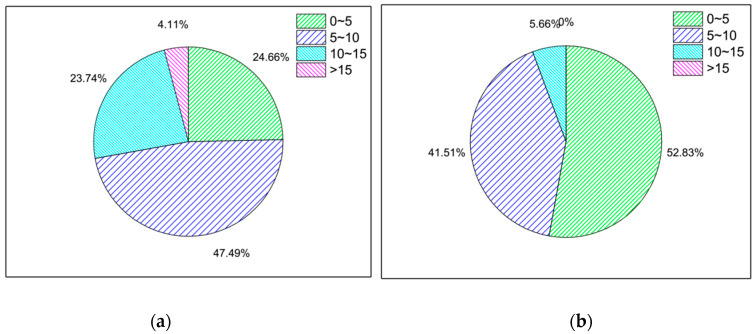
The pie distribution diagram of the standard deviation of passengers’ signal strength during the fine grain time. (**a**) In the bus. (**b**) Out of bus.

During the operation of the bus, the passenger’s position in the bus is generally kept unchanged, unless they need to get off the bus. There is a large relative speed between the mobile device outside the bus and the bus. The moving speed directly affects the stability of the received signal strength. During the fine grain time period, the standard deviation of signal strength received by the Wi-Fi probe is also defined as a feature. The results are shown in Figure 6. The RSSI_ST of the in-bus data was mainly distributed in the 5~10 category, while the RSSI_ST of the out-of-bus data was mainly distributed in the 0~10 category. 

#### 2.3.3. The Sum of Signal Strengths Detected during the Fine-Grained Time Period (RSSI_SUM)

In addition, the sum of signal strength during the fine-grained time period is taken as a feature. The proportion of in-bus data was larger than that of out-bus data in the −750~−500 and −500~−250 categories.

#### 2.3.4. The Length of Time That Data Were Detected during the Fine Grain Time Period (C_T)

**Figure 7:**
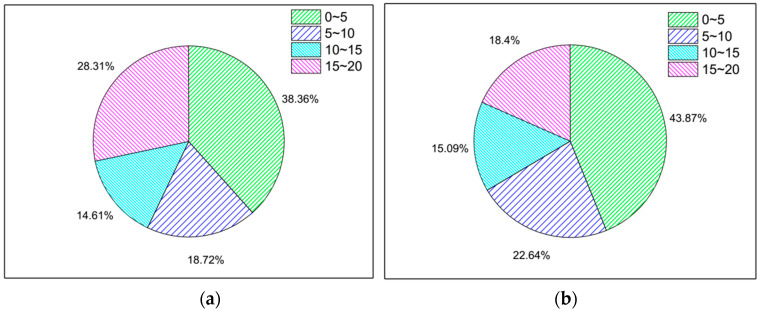
The pie distribution diagram of the length of time are detected within the fine grain time. (**a**) In the bus. (**b**) Out of bus.

In the process of bus movement, the mobile devices outside the bus passes quickly, and the length of time it is detected is obviously different from that inside the bus. Therefore, the length of time detected in the fine-grain time period is selected as a feature. The results are shown in Figure 7.

The C_T of the in-bus data was mainly distributed in the 0~5 s and 15~20 s categories, while the C_T of the out-bus data was mainly distributed in the 0~5 s category. The proportion of in-bus detections was smaller than that of the out-of-bus detections in the 0~5 s and 5~10 s categories, while the proportion of in-bus detections was larger than that of the out-bus detections in the 15~20 s category. The proportion indicate that the scanned time of users by the Wi-Fi probe in the bus is generally longer than that of outside the bus during the fine grain time period.

#### 2.3.5. The Average Number of Times That Data Were Detected during the Fine Grain Time Period (M_AVG)

**Figure 8:**
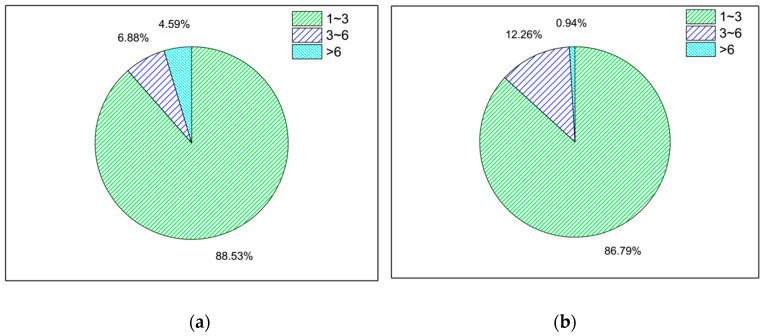
The pie distribution diagram of the average number are detected within the fine grain time. (**a**) In the bus. (**b**) Out of bus.

Same principle as feature C_T, there were significant differences in the average number of probes detected by all mobile devices in and out of the bus during the fine time period. The results are shown in Figure 8.

The M_AVG of the in-bus and out-bus data was mainly distributed in the 1~3 times category. The proportion of in-bus data was larger than that of out-bus data in the 1~3 times category, while the proportion of in-bus data was smaller than that of out-bus data in the 3~6 times category. The proportion of the M_AVG distribution of both in-bus and out-bus environments were more than 80% in the 1~3 times category.

In summary, RSSI_AVG, RSSI_ST, RSSI_SUM, C_T and M_AVG were selected as the features for the machine learning model to learn the rules. We used 70% of the labeled data as a training set and 30% as a validation set.

### 2.4. Data Standardization 

Because the dimension of five features is different, the feature with a small value is weakened when the model is learned. The dimensional feature is transformed into a dimensionless feature to facilitate the comparison and weighting of features of different units, and improve the accuracy of model recognition. Therefore, it is very important to standardize the data before analyzing it. There are many standardization methods: minimum-maximum standardization, Z-Score standardization and Decimal Scaling standardization. In this paper, the minimum–maximum standardization was selected. Since this is a linear transformation, the sorting information of features in each sample is preserved. In fact, the comparison relationship of the features is more useful than the absolute value in the sample. We assume that the set of observed samples is defined as $O=\left\{o_{1},o_{2}\ldots o_{i}\ldots o_{n}\right\}$, where *n* indicates the count of all the samples; each sample is defined as $o=\left\{s_{1},s_{2}\ldots s_{j}\ldots s_{5}\right\}$, $s_{j}$ presents the signal feature value received from the Wi-Fi probes. The processed sample is:

(1) $$o^{\prime }=\mathrm{round}(\frac{x_{\mathrm{ij}}-\min(s_{j})}{\max(s_{j})-\min(s_{j})})$$

where $\max(s_{j})$ and $\min(s_{j})$ are the maximum and minimum of signal feature values in j, respectively. After pretreatment, the values of all the samples are mapped to [−1, 1], which narrows the absolute signal features difference in samples to circumvent the influence of heterogeneous devices.

### 2.5. Machine Learning Techniques

The Wi-Fi probe is used to identify passengers on moving buses, which is affected by many factors, such as the proportion of mobile phone ownership, interference of signals outside the vehicle, complex environment and so on. To achieve high performance, deep learning requires very large data sets. For many applications, such large data sets are not readily available, expensive, and time consuming. For smaller data sets, typical machine learning algorithms are usually better than deep learning. Existing machine learning algorithms are usually divided into supervised learning algorithms and unsupervised learning algorithms. In supervised learning algorithms, the training data and label data are input to the computer. After the computer learns the rules of label data, it calculates the probability that the data belongs to a certain category, and finally gives a result that is closest to the correct result. Since the computer not only has training data but also labels during the learning process, the training effect is positive. 

A machine learning algorithm with supervised learning is adopted in this paper. This kind of algorithm is implemented in three stages [19,20,21]. The first stage, named the training stage, is where data is collected and provided to the classifier to build a model to classify. The second stage, called verifying stage, is where labeled data is collected to verify the accuracy of model. By constantly adjusting the parameters of the first stage, the verification accuracy of this stage reaches its optimal state. The third stage, called predicting stage, is where new data is tested against the model that was built during the first two stages. The machine learning algorithms can be used for classification or regression [22].

#### 2.5.1. Random Forest (RF)

The random forest algorithm is a classification algorithm jointly proposed by Leo and Adele Cutler (2001). It combines Bagging integration, the classification and regression trees (CART) decision tree concept, and the random feature selection concept. Breiman provides mathematical derivations and proofs for random forests, and gives a detailed definition of the random forest concept.

**Figure 9:**
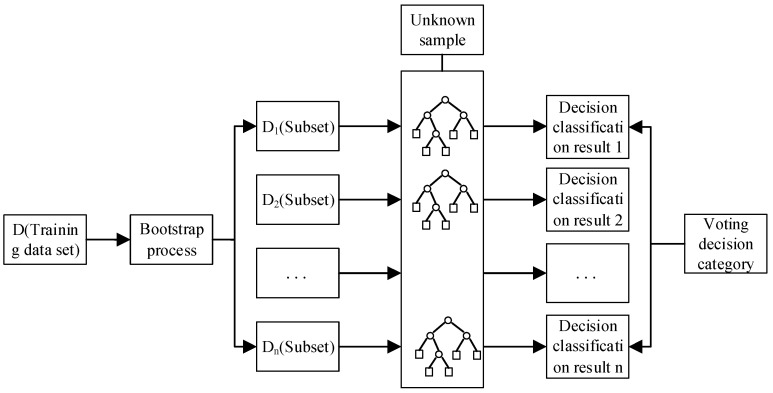
A flow chart of the random forest classification process.

A random forest is a set of classifiers consisting of a series of decision tree classifiers $G(x,\;\theta_{i})\;(i=1,2,\ldots ,n)$, where $\theta_{i}$ is an independent identically distributed random vector, and each decision tree predicts the class attribution of the input variable x. The random forest classification process is as follows (Figure 9):

The tree established by each subset divides the nodes with optimal features. As the partitioning process continues, the samples contained in the branch nodes of the decision tree belong to the same category as much as possible, i.e., the “purity” of the nodes increases. The information gained indicates improvement in the degree of purity of the feature.

(2) $$\mathrm{Ent}(D)=-\sum_{k=1}^{\left|y\right|}p_{k}\mathrm{lo}g_{2}p_{k}$$

where D is the current sample set, $p_{k}$ is the proportion of the k class in D(k=1,2,…,|y|), and Ent(D) is the entropy of D.

(3) $$\mathrm{Gain}(D,a)=\mathrm{Ent}(D)-\sum_{v=1}^{V}\mathrm{Ent}(D^{v})$$

where a is a feature set of D. Feature a has v possible values $\left\{a^{1},a^{2},\ldots ,a^{v}\right\}$. $D^{v}$ is a number of which feature a is $a^{v}$ in set D, $\mathrm{Ent}(D^{v})$ is the entropy of $D^{v}$, and Gain(D,a) is the information gained about feature a for D. In general, the greater the information gained, the greater the “purity increase” obtained by using the feature a to perform the division.

**Figure 10:**
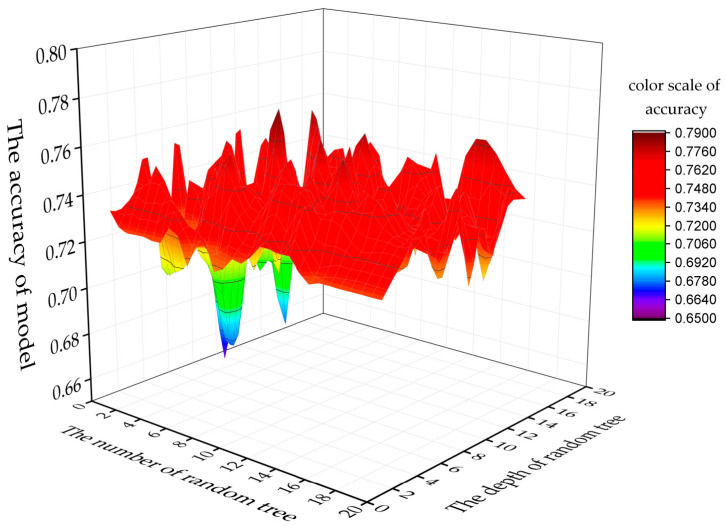
Selection of parameters for the random forest model.

Two of the parameters that can be optimized in Random Forest are the maximum depth of generated tree and number of trees. Figure 10 shows the performance of Random Forest by changing the maximum depth of generated tress. Finally, when the depth (D) was 7 and the number of trees (N) was 11, the model had a high precision position. This result indicates that the optimal depth of trees for 7 trees is 11 with accuracy of 0.7769.

#### 2.5.2. K-Nearest Neighbor (K-NN)

K-nearest neighbor is a simple method that tries to perform classification by calculating the distance between features. The KNN algorithm considers K calibration points. The selection of these points based on selecting the closest K points in the feature space to approximate the position of the user.

**Figure 11:**
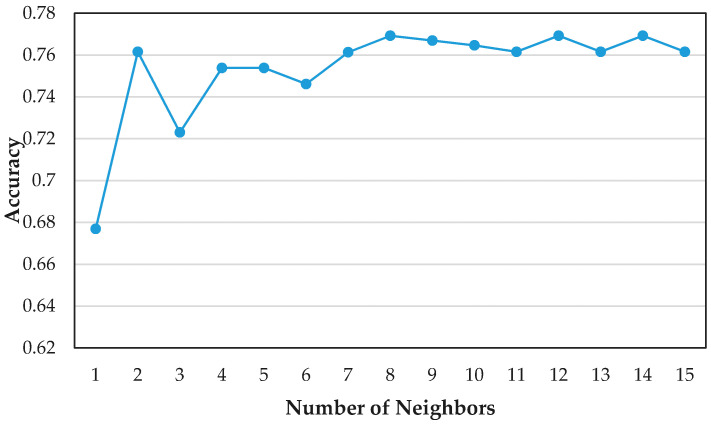
Selection of parameters for the random forest model.

The KNN algorithm calculate the distance between the measured $\bar{y}$ and the vector of the features ${\bar{x}}_{i}$ as shown in the following equation.

(4) $$d(\bar{y}\;-{\bar{x}}_{i})=(\sum_{j=1}^{\left|\bar{y}\right|}{\left|{\bar{y}}_{j}-{\bar{x}}_{\mathrm{ij}}\right|}^{p}{)}^{1/p}$$

where $d(\bar{y}-{\bar{x}}_{i})$ is the distance estimate, ${\bar{x}}_{\mathrm{ij}}$ is the sample average (j indicates the selected feature). In case of p = 1 represents Manhattan norm-distance and in case of p = 2 represents using the Euclidean norm-distance. In this paper, the Euclidean distance is used. Determining the optimal number of neighbors is a challenge task when using KNN for classification. In Figure 11, the prediction accuracy of KNN using different number of neighbors are shown. From the plot, it can be seen that by increasing the number of neighbors from 3 to 8, there is a significant improvement in accuracy. It is worth noting that the best accuracy of 0.7692 is achieved when the number of neighbors is 8.

#### 2.5.3. Support Vector Machines (SVM)

Support Vector Machines are powerful techniques used for classification and data regression. They are used as a non-parametric supervised classifier for pattern recognition problems. SVMs are used in the localization system by training the support features. SVMs analyze the relationship between the trained features and their location as a class. The tested set are taken as an input to SVM that predict the class to which the tested belongs. Before any classification, the signal features are mapped into higher dimensional space using kernel function. The SVM kernel functions K (.,.) is the dot product of two feature vectors x_i_ and x_j_ in some expanded feature space, there are several kernels are proposed by researchers. The four basics kernels as follow: linear, polynomial, sigmoid and radial basis function (RBF). In this research, the linear, polynomial and RBF are used in the following:

Linear:

(5) $$K(x_{i},x_{j})=x_{i}^{T}\cdot x_{j}$$

Polynomial:

(6) $$K(x_{i},x_{j})=(x_{i}^{T}\cdot x_{j}{)}^{n}$$

RBF:

(7) $$K(x_{i},x_{j})=e^{\frac{-{\left\|x_{i}-x_{j}\right\|}^{2}}{2\delta^{2}}}$$

where $\delta^{2}$ is the variance of the Gaussian kernel.

The test feature set with label was input SVM, and the verification accuracy of linear, ploy and RBF was 0.7692, 0.7231 and 0.7615, respectively. 

The classification results of validation set, using machine learning method with five signal features, are shown in Table 2. The random forest showed better performance with an accuracy of 0.7769, so the random forest method was adopted for the prediction feature set.

### 2.6. Proposed Method and Analysis

In this paper, three machine learning algorithms were used to study the data obtained from the in-car and out-car labeled data, and the total number of samples with labels was 434. When the number of neighbors of KNN algorithm is 8, the accuracy of the model starts to stabilize, with an accuracy of 0.7692. The accuracy of SVM algorithm used linear kernel function, polynomial kernel function and Gaussian kernel function were 0.7692, 0.7231 and 0.7615, respectively. The RF algorithm had the best accuracy of 0.7769, in which the depth of trees was 7 and the number of trees was 11. The RF algorithm is only 0.0077 higher than KNN and SVM. Therefore, the method proposed in the following part is analyzed based on the recognition results of the RF algorithm model.

There are 11,044 data samples to be classified, and the recognition results of the machine learning model cannot accurately represent the number of passengers in the bus. There are several reasons: Some people do not carry mobile devices (e.g., the elderly and children), not every bus passenger keeps the Wi-Fi feature of their devices turned on. In addition, the crowded environment in the bus affects the signal transmission and the reception of Wi-Fi probes. Therefore, the recognition result of RF algorithm must be processed by expanding sample to enhance its value.

**Figure 12:**
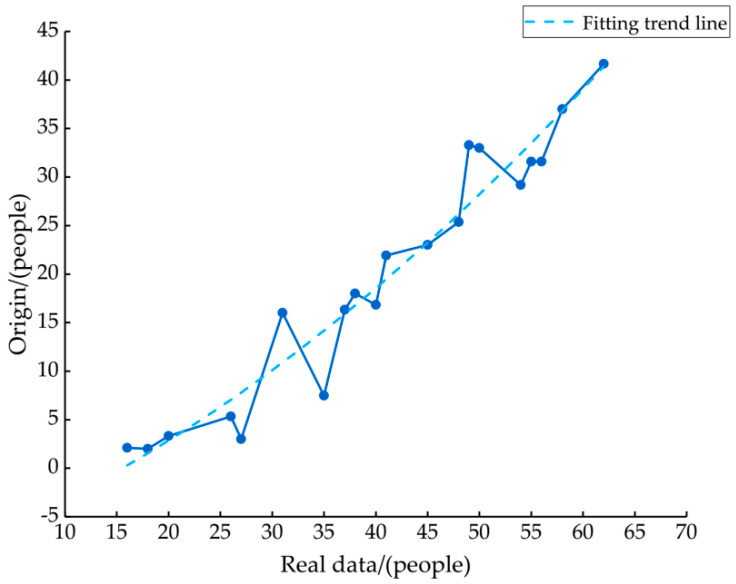
The relationship between real flow and the error between real flow and classification result of RF algorithm.

The fine-grained calculation time of Wi-Fi signal is 20 s, and the classification result of RF algorithm is a fluctuating value between the arrival time of two bus stops. This paper assumes that the arrival time of bus is $t_{i}$. Between $t_{i}$ and $t_{i+1}$, the classified results of the RF algorithm were averaged as the predicted passenger flow of the bus section. There is a large error between the real flow and the predicted flow of RF algorithm, and it is found that the error of the predicted flow increases with the increase in passengers in the bus. Assuming that the prediction error is represented by D, the relationship between D and the real flow has a law: D presents a nonlinear increase with the increase in real flow in section bus line (Figure 12). It is obvious that the classification results of users are greatly affected by the crowded environment in the bus. The wireless signal propagation is carried out by using electromagnetic wave with frequency change, and the dense environment in the bus leads to the attenuation of Wi-Fi signal. In view of this situation, a function is proposed to correct the RF prediction results. A fitting curve is generated between D and the true value by means of unitary quadratic regression. $R^{2}$ represents the goodness of fit coefficient of the curve, which is 0.9375. The function is as follows:

(8) $$D=0.006x^{2}+0.4255x-8.0524$$

(9) D=P−x

where P is the average result of RF algorithm classification in bus section. After the operation, the function expression of P is as follows:

(10) $$P=0.006x^{2}+1.4255x-8.0524$$

This function is called to correct function, which transforms the prediction results of the RF algorithm generating section flow estimate $\tilde{P}$. This result can be used to efficiently estimate the cross sectional flow during bus driving.

## 3. Results 

**Figure 13:**
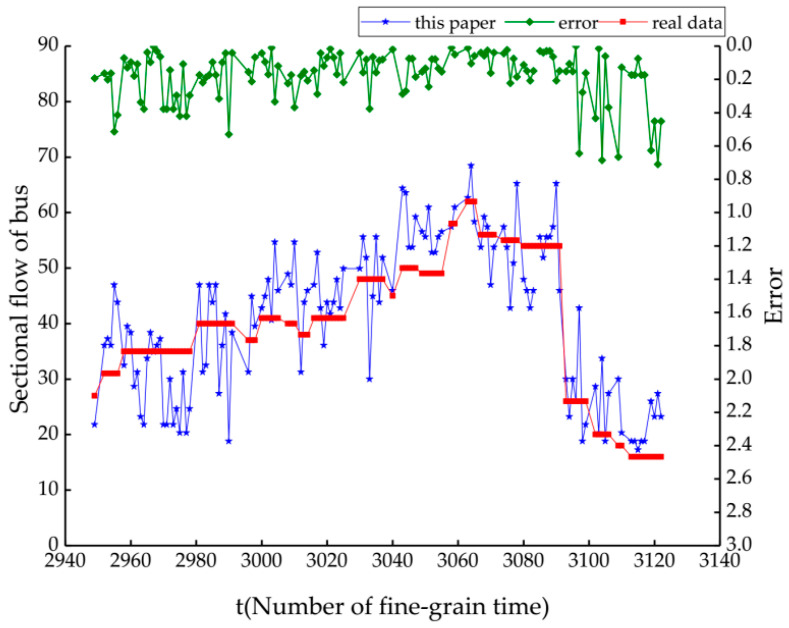
The results of the proposed method.

Through the comparison of the verification accuracy of the three algorithms of RF, KNN and SVM in the previous section, the prediction results of the optimal algorithm were selected to correct. Among them, RF has the highest verification accuracy of 0.7769. We bring the recognition result of the RF algorithm into formula Equations (8)–(10) to obtain the flow estimated value $\tilde{P}$, which is calculated on the fine-grain time of 20 s. Therefore, it is a fluctuating value in the arrival time period of the adjacent bus station, such as the blue symbol line in the Figure 13. The time format of the collected data is ‘yyyy/MM/dd HH:mm:ss’. For the convenience of calculation, the standard time is converted into a fine-grained time period number representation: (HH∗3600+mm∗60+ss)/20. The abscissa in the figure is the fine-grained time number. From this figure, it is seen that the estimated value fluctuates around the true value. The error wave line is the green symbol line and the average error is 0.1338. In this paper, the prediction accuracy of the passenger flow on bus section is 0.8622, which is 0.0893 higher than that of the RF algorithm. This result shows that the accuracy of using the correct function to predict the passenger flow on bus section based on Wi-Fi probe technology is acceptable, but there is still a lot of room for accuracy improvement. Wi-Fi signals are transmitted in the form of electromagnetic waves and are captured by Wi-Fi probes. The intensity of electromagnetic wave is attenuated due to the complex propagation environment of in the bus. The position of Wi-Fi probes in this paper also attenuated this problem. Compared with the short-term passenger flow forecasting approaches [18], the proposed method in this paper reduces the influence of time, space and individual selection behavior, and there is no uncertainty caused by traffic distribution. If only one Wi-Fi probe is installed in the bus [17], the signal strength of the user outside the bus on the side where the probe is located is likely to be stronger than that inside the bus. The experiment designed in this paper arranged six probes evenly distributed in the bus, and recruited 20 volunteers evenly distributed in the bus to collect labeled data. Since the bus itself is a long strip of space, the uniform distribution of Wi-Fi probes avoids the problem of unbalanced signals on both sides of the bus, which is more conducive to extracting the signal characteristics of users. Although the identification results in this paper were not fit enough with the real value, the improved method and reasonable experimental design will achieve better performance in the future work.

This paper innovatively uses Wi-Fi probe technology to collect label data to estimate the cross-section flow of moving buses. However, due to the propagation characteristics of the wireless signal and mobile phone holding rate, there is a large error between the estimated result of the collected signal and the real flow. Therefore, this paper uses the correlation function between the predicted result and real flow to correct the predicted result. Finally, the prediction accuracy of the model reached 0.8622. Rapidly clear of the passenger flow of bus section, the bus operation center can quickly judge whether the upcoming bus is suitable for the waiting passengers at the next bus station, and make reasonable adjustments. This is a study that deserves to be encouraged, especially for buses with a single swipe card.

## 4. Conclusions

Previous studies have mainly investigated pedestrian behavior based on the use of Wi-Fi probes in empty spaces (such as playgrounds), still environments (such as office buildings), and in environments with an isolated signal (such as underground rail environments). There are also a few studies using Wi-Fi probe technology to predict bus passenger flow. In this paper, six Wi-Fi probes were innovatively applied in a bus environment. A total of five characteristics were extracted from mobile terminal data collected on a moving bus. By comparing the verification accuracy of the three algorithms of RF, KNN and SVM, the random forest algorithm was used to train and learn the signal strength law of passengers inside and outside the bus, and then to predict the number of passengers inside the bus. Because the signal strength was affected by the degree of congestion and obstacles, and the collected data is incomplete sample, which reduced the efficacy of RF algorithm. A strain function was proposed to correct the predicted result, and the average predictive error was found to be 0.1338. It is of great value to realize the real-time passenger flow monitoring of single-swipe card. Based on the monitoring of passenger flow, public transport operators formulate dispatch plans in time, and passengers waiting at bus station to decide another alternative travel routes. 

In future work, the predictive accuracy of the model needs to be improved, and the strain function should be adapted to various environments. A more comprehensive Wi-Fi probe survey will be designed to improve the accuracy of passenger flow identification, realize the evaluation of the crowdedness of waiting passengers at bus stations, and improve the operating efficiency of the public transportation system.

## Figures and Tables

**Table 1:** The performance of Wi-Fi probe.

Number	APID	Number of in-Bus Detections	Number of out of Bus Detections
1	2751	26,255	23,338
2	8779	25,853	21,681
3	9345	20,658	23,413
4	2268	8924	22,233
5	2326	25,503	24,002
6	2680	27,382	26,811

**Table 2:** Performance of different machine learning method with label data.

Classifier	Performance
Random forest	0.7769
SVM	0.7692 (linear kernel function)/0.7231 (polynomial kernel function)/0.7615 (RBF)
KNN	0.7692

## Data Availability

The data presented in this study are available on request from the corresponding author. The data are not publicly available due to the data is the privacy of the volunteers.
